# Genome-Wide Identification of MicroRNAs in Leaves and the Developing Head of Four Durum Genotypes during Water Deficit Stress

**DOI:** 10.1371/journal.pone.0142799

**Published:** 2015-11-12

**Authors:** Haipei Liu, Iain R. Searle, Nathan S. Watson-Haigh, Ute Baumann, Diane E. Mather, Amanda J. Able, Jason A. Able

**Affiliations:** 1 School of Agriculture, Food and Wine, Waite Research Institute, University of Adelaide, Glen Osmond, South Australia, Australia; 2 School of Biological Sciences, University of Adelaide, Adelaide, South Australia, Australia; 3 The University of Adelaide-Shanghai Jiao Tong University Joint International Centre for Agriculture & Health, School of Agriculture, Food and Wine, Waite Research Institute, University of Adelaide, Adelaide, South Australia, Australia; 4 Australian Centre for Plant Functional Genomics, School of Agriculture, Food and Wine, University of Adelaide, Adelaide, South Australia, Australia; Cankiri Karatekin University, TURKEY

## Abstract

MicroRNAs (miRNAs) are small non-coding RNAs that play critical roles in plant development and abiotic stress responses. The miRNA transcriptome (miRNAome) under water deficit stress has been investigated in many plant species, but is poorly characterised in durum wheat (*Triticum turgidum* L. ssp. *durum*). Water stress during early reproductive stages can result in significant yield loss in durum wheat and this study describes genotypic differences in the miRNAome between water deficit tolerant and sensitive durum genotypes. Small RNA libraries (96 in total) were constructed from flag leaf and developing head tissues of four durum genotypes, with or without water stress to identify differentially abundant miRNAs. Illumina sequencing detected 110 conserved miRNAs and 159 novel candidate miRNA hairpins with 66 conserved miRNAs and five novel miRNA hairpins differentially abundant under water deficit stress. Ten miRNAs (seven conserved, three novel) were validated through qPCR. Several conserved and novel miRNAs showed unambiguous inverted regulatory profiles between the durum genotypes. Several miRNAs also showed differential abundance between two tissue types regardless of treatment. Predicted mRNA targets (130) of four novel durum miRNAs were characterised using Gene Ontology (GO) which revealed functions common to stress responses and plant development. Negative correlation was observed between several target genes and the corresponding miRNA under water stress. For the first time, we present a comprehensive study of the durum miRNAome under water deficit stress. The identification of differentially abundant miRNAs provides molecular evidence that miRNAs are potential determinants of water stress tolerance in durum wheat. GO analysis of predicted targets contributes to the understanding of genotypic physiological responses leading to stress tolerance capacity. Further functional analysis of specific stress responsive miRNAs and their interaction with targets is ongoing and will assist in developing future durum wheat varieties with enhanced water deficit stress tolerance.

## Introduction

Durum wheat (*Triticum turgidum* L. ssp. *durum*) is the only tetraploid wheat species (2*n* = 4*x* = 28, genomes AABB) grown commercially throughout the world. Water deficit stress is one of the main abiotic factors that cause durum yield loss in Mediterranean environments. Water deficit stress in early reproductive stages has been shown to adversely affect grain yield and biomass through reduced grain number in durum [1]. Nonetheless, Liu et al. also demonstrated that genotypic variation in morphological and physiological responses exists in durum wheat when grown in water limited conditions [1]. Investigating water deficit stress tolerance mechanisms and genotypic differences within a plant species is an important strategy for understanding the basis of stress response and for selection of genotypes with improved water stress tolerance. The genetic mechanism(s) associated with tolerance against abiotic stresses is not well documented in durum wheat, partly because the full genome sequence is still unavailable. Understanding gene regulatory pathways underlying stress responses may lead to new strategies to enhance stress tolerance in durum wheat.

In plants, small non-coding RNAs of 20–24 nucleotides (nts) have been identified as important regulators of genome integrity, virus and pathogen defence, development and importantly, abiotic stress response pathways [2–4]. Small RNAs are broadly divided into microRNAs (miRNAs) and small interfering RNAs (siRNAs). MicroRNAs are global regulators of gene expression mainly through post-transcriptional repression or translational inhibition [5–7]. The general molecular networks related to their complex biogenesis and silencing have now been widely characterised [8–11]. Plant miRNAs control the expression of their targets by binding to imperfect reverse complementary sequences, resulting in degradation and/or translational repression of the cognate target mRNAs [5,11].

Functional analyses of miRNAs and their targets in plants have demonstrated that miRNAs are associated with diverse biological processes including reproductive development and abiotic stress tolerance [12–14]. A large number of studies with different plant models have revealed the up- or down-regulation of certain responsive miRNAs when subjected to various abiotic stresses such as water deficit, salinity, heat and cold stress (Table 1). Stress-responsive miRNAs have displayed different regulation patterns between species. However, some stress responsive miRNAs might also exhibit different expression patterns when comparing genotypes of the same plant species; as shown in cowpea exposed to drought stress [15], wheat exposed to dehydration stress [16] and maize exposed to salt stress [17]. Such genotype-specific responses of miRNA help explain the genetic basis of the phenotypic and physiological differences between genotypes of the same species under stress conditions [15,18]. Furthermore, miRNAs have been shown to display spatio-temporal patterns specific to certain plant tissues, suggesting the involvement of tissue-specific miRNAs in various developmental processes [16–18]. These tissue-specific patterns have been studied in bread wheat [19,20], but not specifically in durum wheat.

**Table 1:** Stress responsive microRNAs and their response to different abiotic treatments in various plant species.

miRNA	Water deficit	Salinity	Heat	Cold	References
miR156	Ath↑, Ttu↑, Osa↓, Tae↑↓, Zma↑	Ath↑, Zma↓	Tae↑	—-	[19,21–26]
miR159	Zma↑, Osa↓, Tae↑	Ath↑, Tae↓	Tae↑	Tae↓	[21,22,24,25,27]
miR160	Peu↑	—-	Tae↑	—-	[25,28]
miR162	Zma↑, Peu↑	Zma↑	—-	—-	[17,21,28]
miR166	Zma↑, Ttu↓, Osa↓	Zma↑	Tae↑	—-	[22,23,25,26]
miR167	Ath↑, Zma↑	Ath↑, Zma↓	Tae↑	Osa↓	[17,21,24,29,30]
miR168	Zma↓, Osa↓, Tae↓	Tae↓, Ath↑, Zma↑	Tae↑	Tae↓	[17,21,22,24,25,27]
miR169	Ath↓**,** Osa↑	Ath↑, Zma↑,	Tae↑	Bdi↑	[17,22,24,31,32]
miR170	Osa↓	Ath↑	—-	—-	[22,24]
miR172	Osa↓, Tae↑	Tae↑	Tae↓	Bdi↑, Tae↑	[22,27,31,32]
miR319	Zma↑, Osa↓	Ath↑	Tae↓		[21,22,24,29]
miR393	Tae↑, Ath↑, Osa↑	Tae↑	Tae↑	Tae↓	[24,27,32,33]
miR395	Zma↓, Osa↑	Zma↑	Tae↑		[17,21,22,29]
miR396	Ath↑, Zma↓, Osa↓, Ttu↓	Ath↑, Zma↓		Ath↑	[17,21–24]
miR397	Osa↓, Tae↓	Tae↓		Bdi↑, Tae↓	[22,27,31,34]
miR398	Zma↑, Ttu↑	Ath↓	Ath↓		[21,23,24,34]
miR399	Zma↓	—-	Tae↑	—-	[21,25]
miR408	Ath↑, Osa↓	—-	—-	Ath↑	[22,24]
miR528	Zma↓, Ttu↓	Zma↑	—-	—-	[17,21,23]
miR529	Osa↓	Tae↓	—-	Tae↑	[22,27]
miR827	Zma↑	—-	Tae↑	—-	[21,25]
miR1029	Tae↑	Tae↓	—-	Tae↑	[27]

Ath, *Arabidopsis thaliana*; Bdi, *Brachypodium distachyon*; Peu, *Populus euphratica*; Ttu, *Triticum turgidum ssp*. *dicoccoides*; Osa, *Oryza sativa*; Zma, *Zea mays*; ↑ = up-regulated; ↓ = down-regulated; —- = not determined

As indicated in Table 1, although numerous miRNAs have been identified in many plant species, including cereals like barley (*Hordeum vulgare*) [35–37], rice (*Oryza sativa*) [38,39], *Brachypodium distachyon* [40,41], and bread wheat (*Triticum aestivum*) [16,19]; only one mature miRNA sequence from *Triticum turgidum* is recorded in the current miRBase v21. A holistic evaluation of cereal miRNA-mediated response mechanisms under stress conditions is far from complete [42], with very little known about miRNAs and their regulatory functions in relation to water deficit stress across multiple durum genotypes.

This study provides insight into miRNA-mediated water deficit stress regulatory pathways, using four Australian durum genotypes with different water deficit sensitivity [1]. Using Illumina sequencing, we identified 110 conserved miRNAs and 159 novel miRNA hairpin candidates in durum. Statistical analysis has revealed 66 conserved water deficit stress responsive miRNA as well as a number of conserved tissue- and genotype-specific miRNAs. In addition, 16 conserved and five novel miRNA hairpins showed contrasting regulatory patterns under water deficit stress between stress tolerant and sensitive genotypes. To our knowledge, this is the first report of water deficit stress responsive miRNAs identified through direct small RNA cloning and sequencing in durum wheat. Furthermore, target prediction and Gene Ontology (GO) analysis suggests that miRNA targets function in a broad range of biological processes such as metabolic process, response to stimuli, reproduction and development. Comparisons of miRNA profiles in different genotypes under stress in combination with the investigation of target functions and their gene ontologies is a promising approach in predicting miRNA-mediated stress signalling mechanisms in durum wheat, which may have the potential for improving abiotic stress tolerance in breeding programs [42,43].

## Results

### Conserved and novel miRNAs in durum discovered using two bioinformatics approaches

To identify conserved and novel miRNAs in durum, 96 sRNA libraries were constructed from flag leaf and head samples from four durum genotypes and sequenced using Illumina high-throughput technology (deposited in NCBI GEO Database, accession number GSE69339). Approximately 623.4 million reads were obtained from these 96 libraries which represent 16 biological groups (four durum genotypes from each of two tissue types and two water deficit stress treatment groups with six biological replicates in each) (S1 Table). The average number of reads per library was approximately 6.5 million.

For conserved miRNA identification, Approach #1 was developed using CLC Genomics Workbench v7.0 (CLC Bio, Denmark). Approximately 602.1 million reads (that is, 6.3 million per library on average) were obtained after removing low quality sequences, those without inserts, or those with adapter contaminants or lengths outside of the 15–50 nt range. Among the trimmed reads, approximately 301.8 million non-redundant unique small RNA reads were obtained. The most abundant sRNA reads were 21–24 nucleotides (nt), with 24 nt reads being the most common in length (S1 Fig). Unique, mature plant miRNA sequences from nine common monocot and dicot species (*Triticum aestivum*, *Triticum turgidum*, *Brachypodium distachyon*, *Zea mays*, *Oryza sativa*, *Hordeum vulgare*, *Sorghum bicolor*, *Arabidopsis thaliana*, and *Glycine max*) deposited in miRBase were used as references to identify conserved miRNAs in durum wheat allowing a maximum of two mismatches in alignment. Approximately 21.6 million sRNA reads were annotated in 96 libraries, and nearly 2 million annotated tags matched 110 conserved miRNAs in the nine selected plant species (S2 Table).

For novel miRNA identification, a customised bioinformatics approach (Approach #2) was developed. Putative miRNA hairpins were identified using the latest International Wheat Genome Sequencing Consortium’s (IWGSC) Chromosomal Survey Sequences (CSS) of bread wheat [44], due to the limited availability of durum wheat sequence. This process resulted in the identification of an initial set of 6,643 loci representing 3,421 non-redundant sequences. Of these non-redundant sequences, 2,710 sequences passed checks by RNAFold and miRcheck, which satisfied *in silico* requirements of the biogenesis pathway of miRNAs in plants. Of these 2,710 candidate miRNA hairpin sequences, 237 matched the expectations for a true miRNA in terms of their read coverage profile (Category A) using three Boolean metrics as described in the Materials and Methods. Of these, 78 contained an exact match to at least one known mature miRNA from miRBase (Table 2), while the remaining 159 putative novel miRNAs had no match to any known mature miRNAs in the miRBase (S3 Table).

**Table 2:** Summary of putative miRNA hairpins in durum wheat small RNA libraries.

Category	≥95% Strand Bias	≥95% reads in one of the terminal 50bp	≤5% reads in loop region	Number of miRNA hairpins	Number of hairpins with known miRNA	Number of hairpins with putative novel miRNA
A	Y	Y	Y	237	78	159
B	Y	Y	N	96	33	63
C	Y	N	Y	100	55	45
D	Y	N	N	322	72	250
E	N	Y	Y	93	4	89
F	N	Y	N	161	17	144
G	N	N	Y	145	17	128
H	N	N	N	1556	134	1422
Total				2710	410	2300

All putative miRNA hairpin sequences were classified into one of eight categories (A-H, where category A candidates have a read coverage profile matching the expectations for a true miRNA) using 3 Boolean metrics based on the read coverage profile: 1) If ≥ 95% of the reads mapped to one strand of the hairpin; 2) If ≥ 95% of the reads mapped to one of the terminal 50 bp of the hairpin; 3) If ≤ 5% of the reads mapped to the loop region of the hairpin.

### Some conserved durum miRNAs are genotype- or tissue-specific regardless of water-deficit stress (Approach #1)

**Fig 1:**
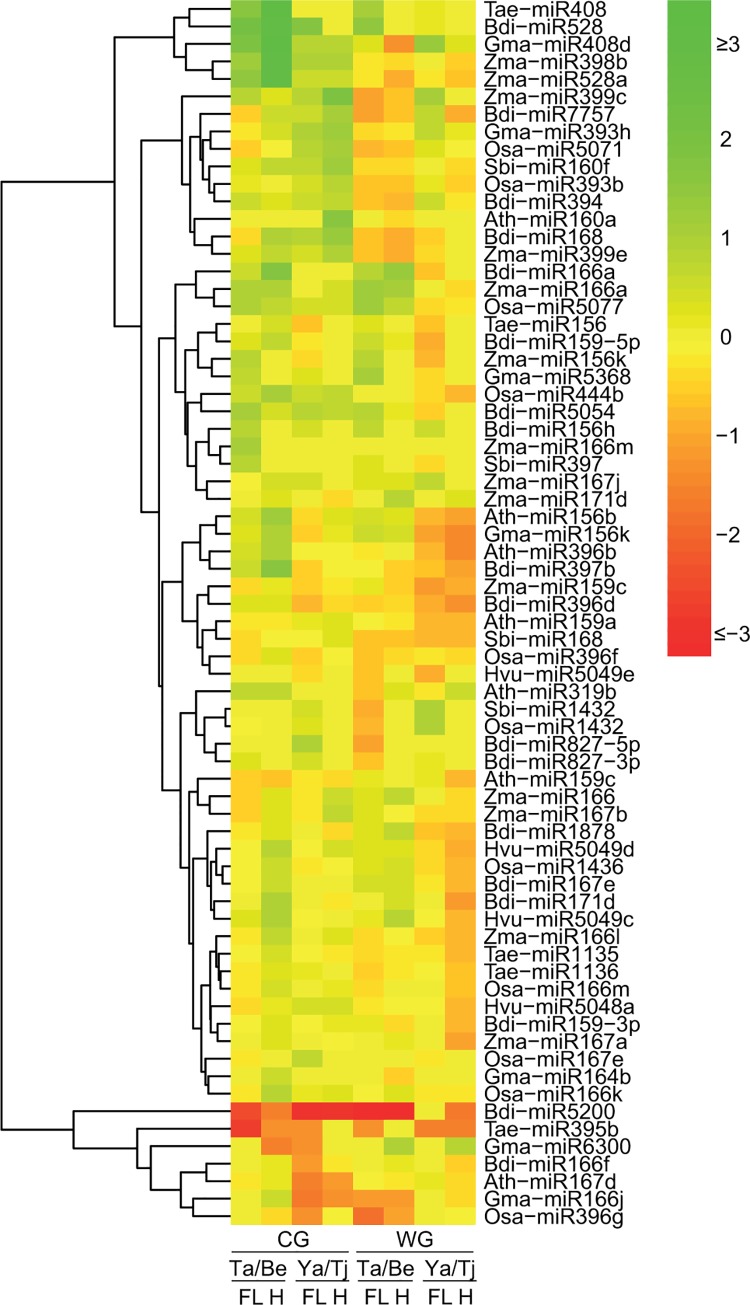
Heat-map showing differential expression patterns of conserved miRNAs between different genotypes revealed by high-throughput sequencing. The colour scale is based on the log2 value of the fold-change of the water deficit stress tolerant variety (Tamaroi or Yawa) libraries compared to the water deficit stress sensitive variety (EGA Bellaroi or Tjilkuri) libraries. Log2 value = log2 (RPM of miRNA reads in Tamaroi library/RPM of miRNA reads in EGA Bellaroi library) or log2 (RPM of miRNA reads in Yawa library/RPM of miRNA reads in Tjilkuri library). The red colour indicates that the miRNA was more abundant in the water deficit stress sensitive variety; while the green colour indicates that the miRNA was more abundant in the water deficit stress tolerant variety. CG = Control group; WG = Water deficit stress group; FL = Flag leaf samples; H = Head samples; Be = EGA Bellaroi; Ta = Tamaroi; Tj = Tjilkuri; Ya = Yawa; Ath = *Arabidopsis thaliana*; Bdi = *Brachypodium distachyon*; Hvu = *Hordeum vulgare*; Gma = *Glycine max*; Osa = *Oryza sativa*; Sbi = *Sorghum bicolor*; Tae = *Triticum aestivum*; Zma = *Zea mays*.

Differential miRNA expression profiles were observed between the water deficit stress sensitive (EGA Bellaroi and Tjilkuri) and tolerant (Tamaroi and Yawa) genotypes across both treatments. Comparisons were made between Tamaroi versus EGA Bellaroi, and Yawa versus Tjilkuri, separately, based on their breeding history and genetic background. A total of 70 miRNAs were differentially expressed between different durum genotypes (Fig 1). Among these miRNAs, four groups displayed interesting expression patterns between the water deficit stress tolerant and the sensitive genotypes (Table 3): I) miRNAs predominantly expressed in water deficit tolerant genotypes under both treatments (7 miRNAs); II) miRNAs predominantly expressed in the water deficit sensitive genotypes under both treatments (5 miRNAs); III) miRNAs predominantly expressed in the water deficit sensitive genotypes under water deficit stress treatment, but predominantly expressed in the water deficit tolerant genotypes under the control treatment (9 miRNAs); IV) miRNAs predominantly expressed in the water deficit tolerant genotypes under the water deficit stress treatment, but predominantly expressed in the water deficit sensitive genotypes under the control treatment (1 miRNA). For example, in group I, the expression level of Osa-miR5077 was more abundant in Tamaroi compared to EGA Bellaroi in both tissues under both treatments (1.95 fold in control flag leaf libraries, 2.41 fold in water deficit flag leaf libraries, 1.58 fold in control head libraries and 1.60 fold in water deficit head libraries respectively) (Table 3). In group III, Osa-miR5071 was more abundant in Yawa compared to Tjilkuri in the control treatment libraries (1.78 fold in the flag leaf and 2.10 fold in the developing head, respectively); but was more abundant in EGA Bellaroi compared to Tamaroi in the water deficit treatments (1.78 fold in the flag leaf and 1.56 fold in the developing head, respectively) (Table 3).

**Table 3:** Genotype-specific durum miRNAs showed four different regulation patterns to water deficit stress.

Name	Resource species in miRBase	Group	CG				WG			
			Ta vs. Be		Ya vs. Tj		Ta vs. Be		Ya vs. Tj	
			FL	H	FL	H	FL	H	FL	H
miR160f	Sbi	I		**1.54**	**1.60**	**2.27**				
miR166a	Zma	I	**1.90**	**1.92**			**2.30**	**2.21**		
miR393h	Gma	I			**2.00**	**2.23**			**1.60**	
miR408	Tae	I	**3.04**	**17.98**			**2.18**			
miR5054	Bdi	I	**2.03**		**1.70**	**1.55**	**1.75**			
miR5077	Osa	I	**1.95**	**1.58**			**2.41**	**1.60**		
miR528	Bdi	I	**4.42**	**9.23**	**3.34**		**2.86**			
miR166j	Gma	II			3.22	2.53	2.25	2.26		
miR395b	Tae	II	6.65	2.51	2.54		2.59		2.96	3.03
miR396d	Bdi	II			1.64				1.88	2.50
miR396g	Osa	II			2.59		3.46	2.04		
miR5200	Bdi	II	5.88	2.88	9.21	7.74	12.94	12.69		3.12
miR156k	Gma	III		**1.95**					2.01	2.81
miR168	Bdi	III		**1.91**	**1.72**	**2.44**	1.59	1.89		
miR319b	Ath	III	**1.53**	**1.55**			1.52			
miR393b	Osa	III				**1.81**	1.56	1.59		
miR398b	Zma	III	**2.42**	**10.11**	**1.95**	**1.97**				
miR399e	Zma	III		**1.60**		**1.87**	1.58	1.89		
miR444b	Osa	III		**2.03**		**1.65**				1.68
miR5071	Osa	III			**1.78**	**2.10**	1.78	1.56		
miR528a	Zma	III	**2.84**	**9.75**				1.88		1.55
miR6300	Gma	IV		2.95	2.48			**1.85**		**1.74**

Fold changes have been determined by comparing the reads per million (RPM) between Tamaroi and EGA Bellaroi, Yawa and Tjilkuri in different treatment groups, and different tissues. Bold fold change values indicate that the miRNA reads were more abundant in the water deficit stress tolerant genotypes (Tamaroi or Yawa), while unbolded fold change values indicate that miRNA reads were more abundant in the water deficit stress sensitive genotypes (EGA Bellaroi or Tjilkuri). Blanks indicate that the fold change is either under 1.5 or the fold change is undetermined due to low abundance in the sequencing libraries. Four groups of miRNAs showed interesting expression patterns between the water deficit stress tolerant/sensitive genotypes: I) miRNAs predominantly expressed in the water deficit tolerant genotypes under both treatments; II) miRNAs predominantly expressed in the water deficit sensitive genotypes under both treatments; III) miRNAs predominantly expressed in the water deficit sensitive genotypes under the water deficit stress treatment but predominantly expressed in the water deficit tolerant genotypes under the control treatment; IV) miRNAs predominantly expressed in the water deficit tolerant genotypes under the water deficit stress treatment but predominantly expressed in the water deficit sensitive genotypes under the control treatment. CG = Control group; WG = Water deficit stress group; FL = Flag leaf libraries; H = Head libraries; Be = EGA Bellaroi; Ta = Tamaroi; Tj = Tjilkuri; Ya = Yawa; Ath = *Arabidopsis thaliana*; Bdi = *Brachypodium distachyon*; Gma = *Glycine max*; Osa = *Oryza sativa*; Sbi = *Sorghum bicolor*; Tae = *Triticum aestivum*; Zma = *Zea mays*.

**Fig 2:**
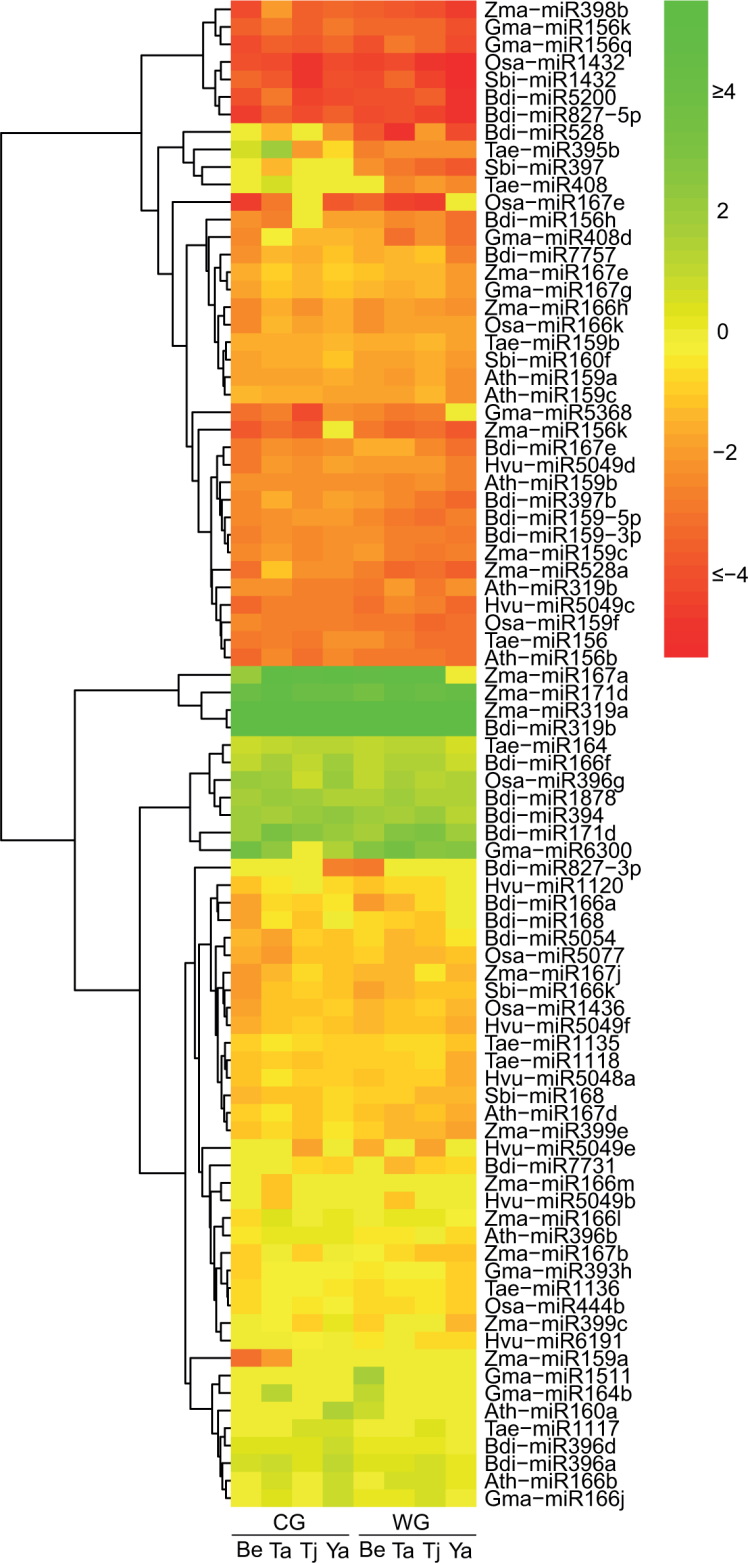
Heat-map showing differential expression patterns of conserved miRNAs between different tissues revealed by high-throughput sequencing. The colour scale is based on the log2 value of the fold-change of the developing head libraries compared to the flag leaf libraries in four durum genotypes under different water treatments. Log2 value = log2 (RPM of miRNA reads in head libraries/RPM of miRNA reads in flag leaf libraries). The red colour indicates that the miRNA was more abundant in the flag leaf libraries; while the green colour indicates that the miRNA was more abundant in the developing head libraries. CG = Control group; WG = Water deficit stress group; Be = EGA Bellaroi; Ta = Tamaroi; Tj = Tjilkuri; Ya = Yawa; Ath = *Arabidopsis thaliana*; Bdi = *Brachypodium distachyon*; Hvu = *Hordeum vulgare*; Gma = *Glycine max*; Osa = *Oryza sativa*; Sbi = *Sorghum bicolor*; Tae = *Triticum aestivum*; Zma = *Zea mays*.

A comparison between all flag leaf and developing head samples identified miRNAs displaying differential abundance between different tissues, irrespective of genotype and treatment. While a total of 110 conserved miRNAs were identified in all sRNA libraries, 86 miRNAs were differentially abundant between flag leaf tissue and the developing head tissue (Fig 2). A total of nine miRNAs were predominantly expressed in the developing head tissue in all four durum genotypes across both treatments while 37 miRNAs were predominantly expressed in the flag leaf tissue (Table 4). For example, Bdi-miR171d was more abundant (from 2.99 to 9.35 fold greater) in the developing head libraries compared to the flag leaf libraries in the four durum genotypes irrespective of the treatment. In contrast, Tae-miR156 was more abundant (from 4.60 to 8.66 fold greater) in the flag leaf libraries compared to the developing head libraries in the four durum genotypes irrespective of the treatment (Table 4).

**Table 4:** Durum miRNAs showed tissue-specific expression profiles regardless of water deficit stress.

Name	Resource species in miRBase	H vs. FL							
		CG				WG			
		Be	Ta	Tj	Ya	Be	Ta	Tj	Ya
miR164	Tae	**1.77**	**2.04**	**2.20**	**2.23**	**2.13**	**2.51**	**2.23**	**1.57**
miR166f	Bdi	**2.00**	**3.06**	**1.98**	**3.50**	**2.15**	**2.68**	**2.71**	**1.70**
miR171d	Bdi	**3.66**	**9.35**	**6.06**	**4.48**	**2.99**	**6.81**	**8.08**	**3.43**
miR171d	Zma	**15.80**	**31.10**	**16.08**	**17.38**	**10.62**	**23.51**	**15.62**	**23.46**
miR1878	Bdi	**3.24**	**4.40**	**3.45**	**2.75**	**2.89**	**3.81**	**2.94**	**2.61**
miR319a	Zma	**38.68**	**60.19**	**51.90**	**58.11**	**61.28**	**57.17**	**41.15**	**54.74**
miR319b	Bdi	**86.21**	**106.61**	**90.62**	**97.54**	**111.48**	**102.45**	**63.82**	**71.59**
miR394	Bdi	**3.96**	**3.36**	**4.60**	**4.99**	**3.86**	**3.36**	**4.33**	**2.48**
miR396g	Osa	**4.27**	**3.98**	**1.73**	**4.19**	**1.95**	**3.30**	**2.29**	**2.64**
miR1118	Tae	2.40	1.82	2.18	1.90	1.95	2.04	1.57	2.90
miR1432	Osa	15.95	18.01	27.73	18.03	23.05	17.16	28.02	102.93
miR1432	Sbi	10.47	13.31	27.77	15.61	19.83	9.93	20.66	76.54
miR1436	Osa	3.27	2.09	2.15	1.87	2.60	2.37	1.94	2.64
miR156	Tae	7.89	5.98	7.43	4.60	5.09	6.08	8.34	8.66
miR156b	Ath	9.86	5.49	8.29	5.16	7.06	7.55	6.98	8.90
miR156k	Gma	12.39	7.80	11.52	7.19	9.61	9.91	10.84	15.12
miR156q	Gma	17.46	11.48	13.38	10.47	15.01	7.87	10.07	19.20
miR159-3p	Bdi	5.96	4.59	5.64	4.72	4.65	6.21	6.83	7.23
miR159-5p	Bdi	5.81	4.42	4.31	3.95	5.31	7.18	8.72	6.90
miR159a	Ath	3.65	3.74	3.51	3.17	3.58	3.91	3.05	5.01
miR159b	Ath	4.68	4.59	4.56	4.43	4.65	5.88	4.44	6.16
miR159b	Tae	3.20	3.07	2.82	2.60	3.00	3.21	2.63	3.47
miR159c	Ath	2.65	2.86	2.97	3.27	3.48	3.78	2.44	4.51
miR159c	Zma	5.85	4.34	5.45	4.58	4.03	5.96	7.11	6.06
miR159f	Osa	5.53	6.59	6.36	6.51	7.92	7.56	10.04	8.70
miR160f	Sbi	1.77	2.04	2.20	2.23	2.13	2.51	2.23	1.57
miR166h	Zma	5.39	2.95	5.02	2.82	4.45	3.36	3.97	5.10
miR166k	Sbi	3.86	2.12	2.01	2.17	3.31	2.60	2.26	2.23
miR166k	Osa	5.58	2.67	3.43	3.13	4.93	3.62	3.60	3.62
miR167d	Ath	2.05	1.52	2.15	1.63	2.29	2.62	2.23	2.99
miR167e	Bdi	7.97	4.89	5.28	4.85	3.15	3.25	5.49	8.05
miR167e	Zma	3.09	2.02	2.53	1.92	2.25	2.64	2.73	4.02
miR167g	Gma	3.35	2.41	2.47	2.12	2.76	2.92	2.75	3.98
miR168	Sbi	2.53	2.14	2.34	1.65	2.01	1.95	2.46	2.51
miR319b	Ath	4.66	4.61	6.38	6.53	7.90	4.25	7.84	4.81
miR397b	Bdi	5.57	2.82	4.49	3.34	4.10	5.51	7.14	9.77
miR398b	Zma	18.22	4.36	10.95	10.83	11.54	13.51	16.78	24.72
miR399e	Zma	2.31	1.72	2.20	1.52	2.03	2.43	2.54	3.61
miR5049c	Hvu	10.42	6.64	6.85	6.13	9.31	5.72	6.42	10.15
miR5049d	Hvu	7.61	4.05	4.94	3.43	3.95	4.00	4.23	6.36
miR5049f	Hvu	3.09	2.31	1.88	2.16	2.63	2.22	2.15	3.24
miR5077	Osa	3.11	3.82	2.18	2.21	1.80	2.72	2.44	2.23
miR5200	Bdi	16.15	7.91	21.11	17.74	15.67	15.37	11.86	36.72
miR528a	Zma	8.10	2.36	4.95	5.05	6.43	9.92	9.19	12.06
miR7757	Bdi	5.02	2.49	3.01	2.11	3.21	2.44	2.23	6.69
miR827-5p	Bdi	23.90	11.91	18.34	12.09	18.30	16.37	22.78	48.05

Fold changes have been determined by comparing the RPM between flag leaf libraries and head libraries in four durum wheat genotypes under different water treatments. Bold fold change values indicate that the miRNA reads were more abundant in the head libraries (nine miRNAs), while unbolded fold change values indicate that the miRNA reads were more abundant in flag leaf libraries (37 miRNAs). CG = Control group; WG = Water deficit stress group; FL = Flag leaf libraries; H = Head libraries; Be = EGA Bellaroi; Ta = Tamaroi; Tj = Tjilkuri; Ya = Yawa; Ath = *Arabidopsis thaliana*; Bdi = *Brachypodium distachyon*; Gma = *Glycine max*; Osa = *Oryza sativa*; Sbi = *Sorghum bicolor*; Tae = *Triticum aestivum*; Zma = *Zea mays*.

### Water deficit stress-responsive conserved miRNAs in durum (Approach #1)

**Fig 3:**
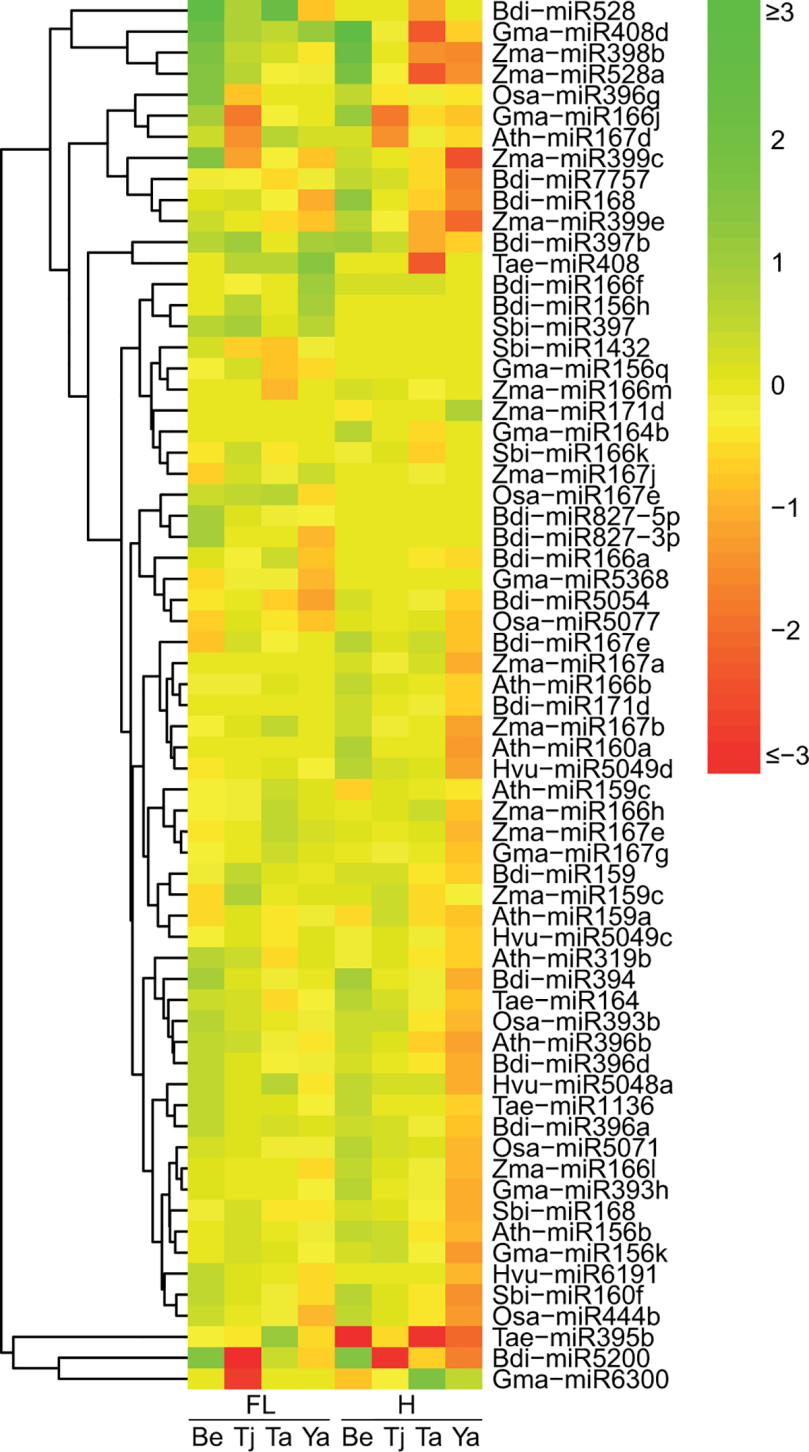
Heat-map showing expression patterns of water deficit stress responsive conserved miRNAs revealed by high-throughput sequencing. The colour scale is based on the log2 value of the fold-change of the water deficit stress treatment libraries compared to the control treatment libraries in four durum genotypes. Log2 value = log2 (RPM of miRNA reads in water deficit stress libraries/RPM of miRNA reads in to control libraries). The red colour indicates that the miRNA was more abundant in the control libraries; while the green colour indicates that the miRNA was more abundant in the water deficit treatment libraries. CG = Control group; WG = Water deficit stress group; FL = Flag leaf samples; H = Head samples; Be = EGA Bellaroi; Ta = Tamaroi; Tj = Tjilkuri; Ya = Yawa; Ath = *Arabidopsis thaliana*; Bdi = *Brachypodium distachyon*; Hvu = *Hordeum vulgare*; Gma = *Glycine max*; Osa = *Oryza sativa*; Sbi = *Sorghum bicolor*; Tae = *Triticum aestivum*; Zma = *Zea mays*.

Differential expression of conserved miRNAs were found between water deficit stressed and corresponding control libraries in both the flag leaf and developing head tissues of each durum genotype. Using the criteria described in the Materials and Methods, 66 conserved mature miRNAs were determined to be water deficit stress-responsive miRNAs (Fig 3 and S4 Table).

**Fig 4:**
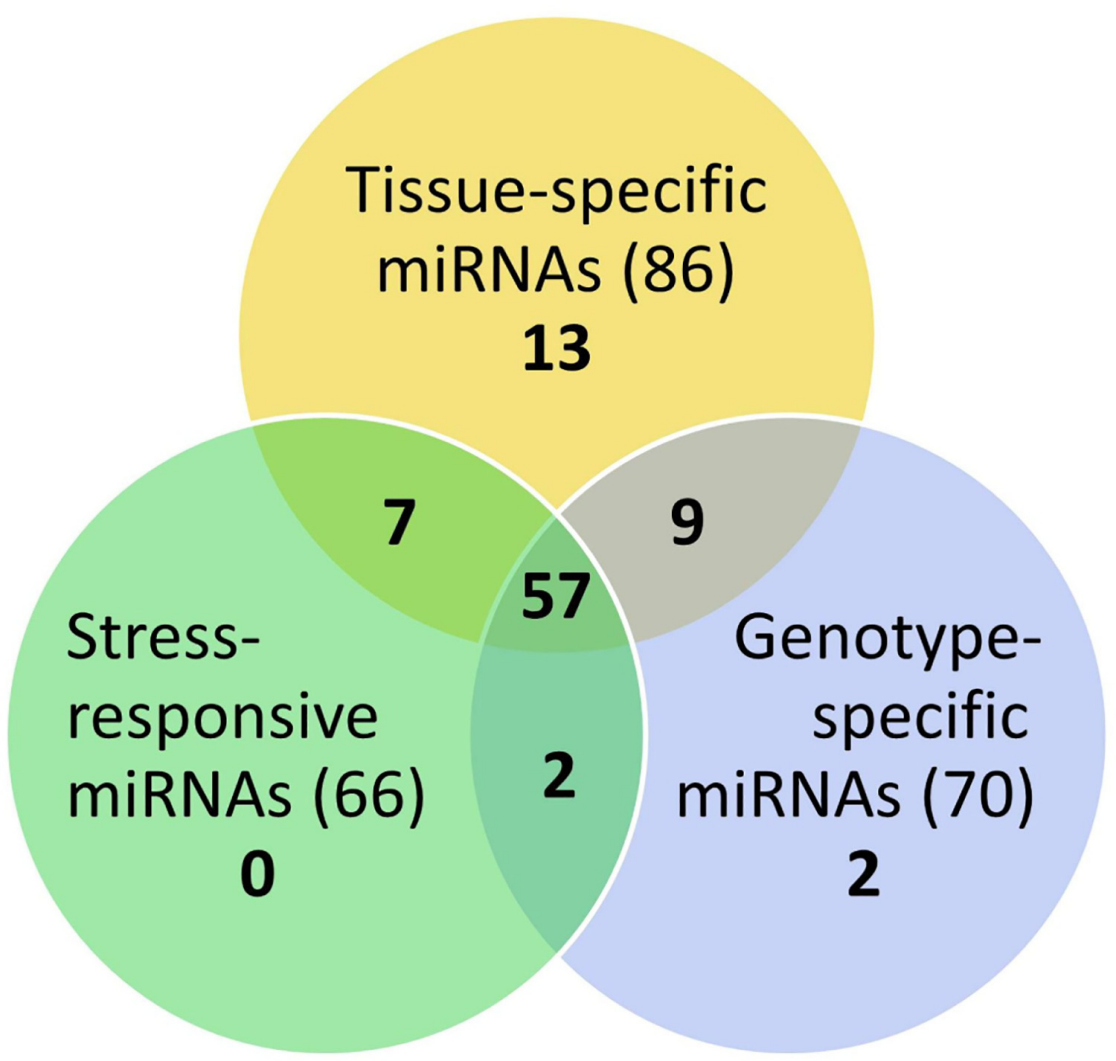
Venn diagram of all differentially expressed conserved microRNAs identified through Approach #1. The number of microRNAs that were differentially abundant in each category is indicated. A total of 57 conserved miRNAs were identified as being responsive to water deficit stress, as well as being differentially abundant across different genotypes and tissue types.

Hierarchical clustering of the water deficit stress-responsive miRNAs illustrated that several miRNAs showed different regulation patterns under water deficit stress between stress tolerant and sensitive genotypes (Fig 3), whereas certain miRNAs showed the same regulation patterns (e.g. Gma-miR408d was up-regulated under stress of all four durum genotypes in the flag leaf tissues). More interestingly, a small number of stress responsive miRNAs showed up-regulation in water deficit stress sensitive genotypes while those same miRNAs were down-regulated in the tolerant genotypes. For example, in the developing head libraries, Bdi-miR7757 was up-regulated in the sensitive genotypes (EGA Bellaroi and Tjilkuri), but was down-regulated in the tolerant genotypes (Tamaroi and Yawa) (Fig 3 and S4 Table). Moreover, some miRNAs responded to water deficit stress only in stress tolerant or sensitive genotypes. In the head libraries, there were 26 miRNAs that were only down-regulated in the stress tolerant genotype Yawa, but not in the stress sensitive genotypes EGA Bellaroi or Tjilkuri (Fig 3). In summary, through further analysing the differentially expressed miRNAs identified through Approach #1, 57 conserved miRNAs were identified as being responsive to water deficit stress, as well as being differentially abundant across different genotypes and tissue types (Fig 4).

### Conserved and novel miRNA hairpins showed inverted expression profiles in response to water deficit stress across genotypes (Approach #2)

**Fig 5:**
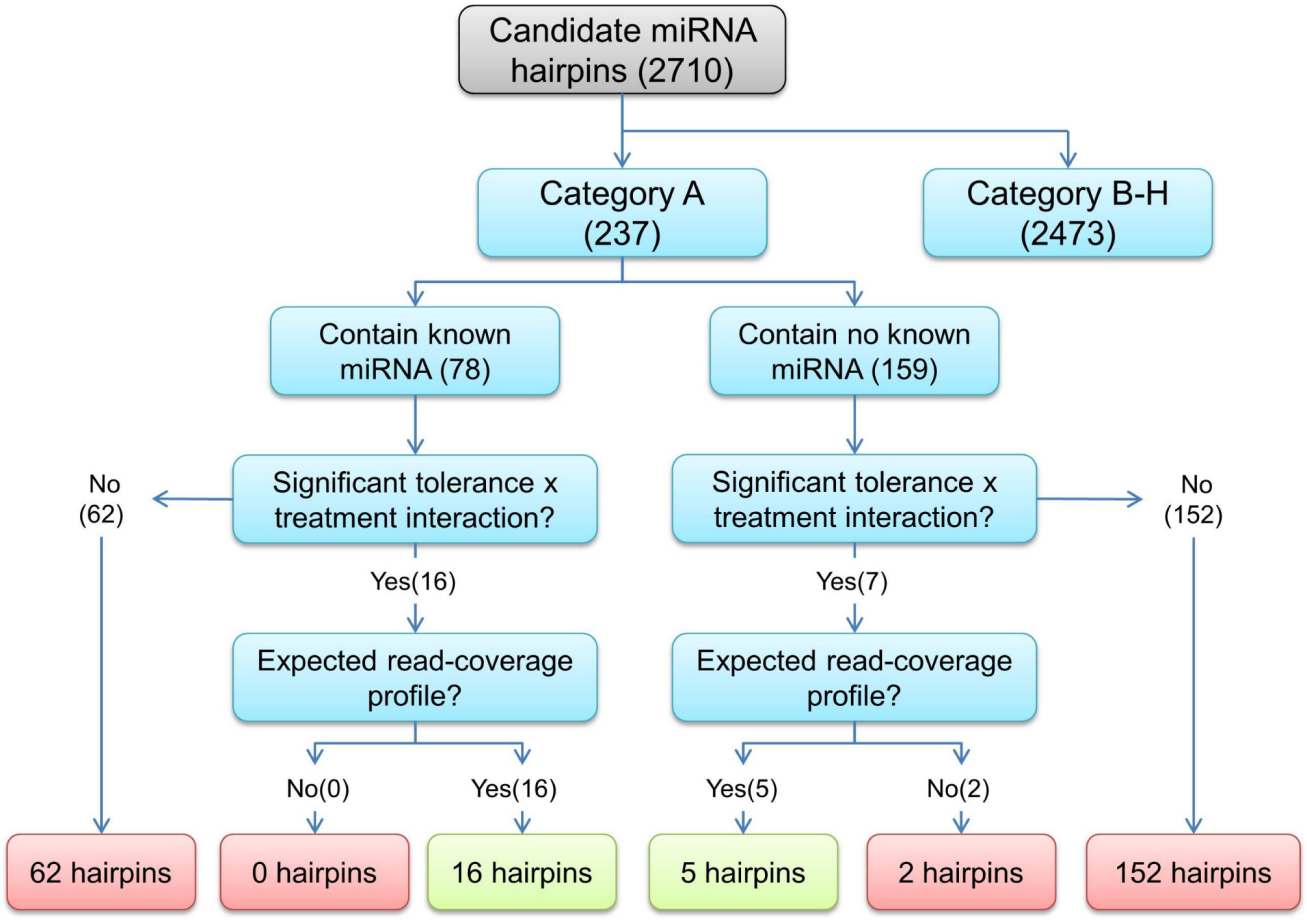
A schematic representation displaying the breakdown of water deficit stress responsive miRNA hairpins identified. A total of 16 hairpins have a significant tolerance × treatment interaction term and contain at least one perfect alignment to a known mature miRNA, while five hairpins have a significant tolerance × treatment interaction term but do not contain a perfect alignment to any known mature miRNAs.

**Fig 6:**
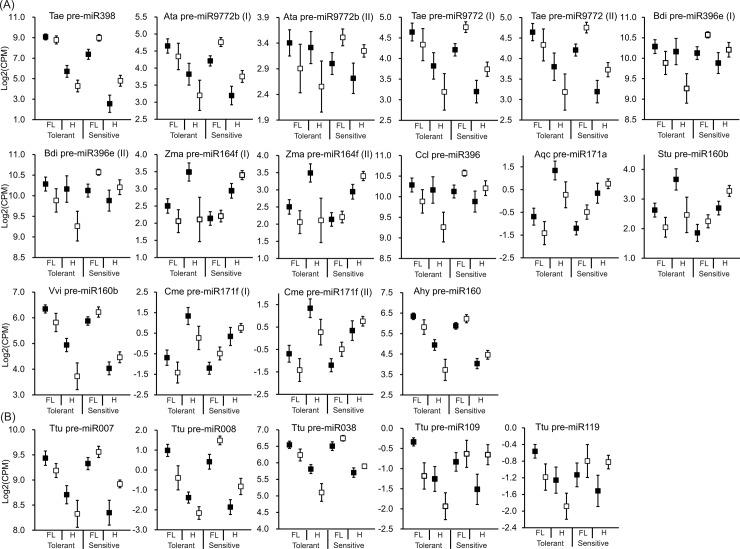
Expression profiles of stress responsive miRNA hairpins showing inverted regulatory patterns between stress tolerant/sensitive genotypes. In (A) 16 conserved miRNA hairpins representing 11 conserved miRNAs are shown, while in (B) five novel miRNA hairpins representing four conserved miRNAs are displayed. The log2 value of normalised reads for each miRNA hairpin is represented as counts per million (CPM). ■ = Control group; □ = Water deficit stress group. Tae = *Triticum aestivum*; Ata = *Aegilops tauschii*; Bdi = *Brachypodium distachyon*; Zma = *Zea mays*; Ccl = *Citrus clementina*; Aqc = *Aquilegia coerulea*; Stu = *Solanum tuberosum*; Vvi = *Vitis vinifera*; Cme = *Cucumis melo*; Aty = *Arachis hypogaea*; Tolerant = Stress tolerant genotypes (Tamaroi and Yawa); Sensitive = Stress sensitive genotypes (EGA Bellaroi and Tjilkuri); I and II denotes two different hairpins representing the same conserved miRNA.

Using the Limma Bioconductor package [45,46], 23 of the 237 putative miRNA hairpins in Category A were found to have a significant tolerance × treatment interaction term. On manual inspection of the miRNA hairpin read-coverage profiles in Category A, 21 of these 23 miRNA hairpins represent strong candidates as they have good read-coverage signatures (Fig 5). Of these 21 candidates, we determined that 16 perfectly matched at least one known mature miRNA in the miRBase, with some hairpins matching to the same conserved miRNA (Fig 6A). The remaining five novel candidate miRNA hairpins, representing four mature novel miRNAs, do not contain a perfect alignment to any known mature miRNAs (Fig 6B and S2 Fig). For example, miRNA hairpin Ttu pre-miR008 representing Ttu-miR008 was down-regulated in both flag leaf and developing head tissues under water deficit stress in the stress tolerant genotypes (Tamaroi and Yawa), but was up-regulated in the stress sensitive genotypes (EGA Bellaroi and Tjilkuri).

### Validation of differentially expressed miRNAs in durum wheat by quantitative real-time PCR (qPCR)

**Fig 7:**
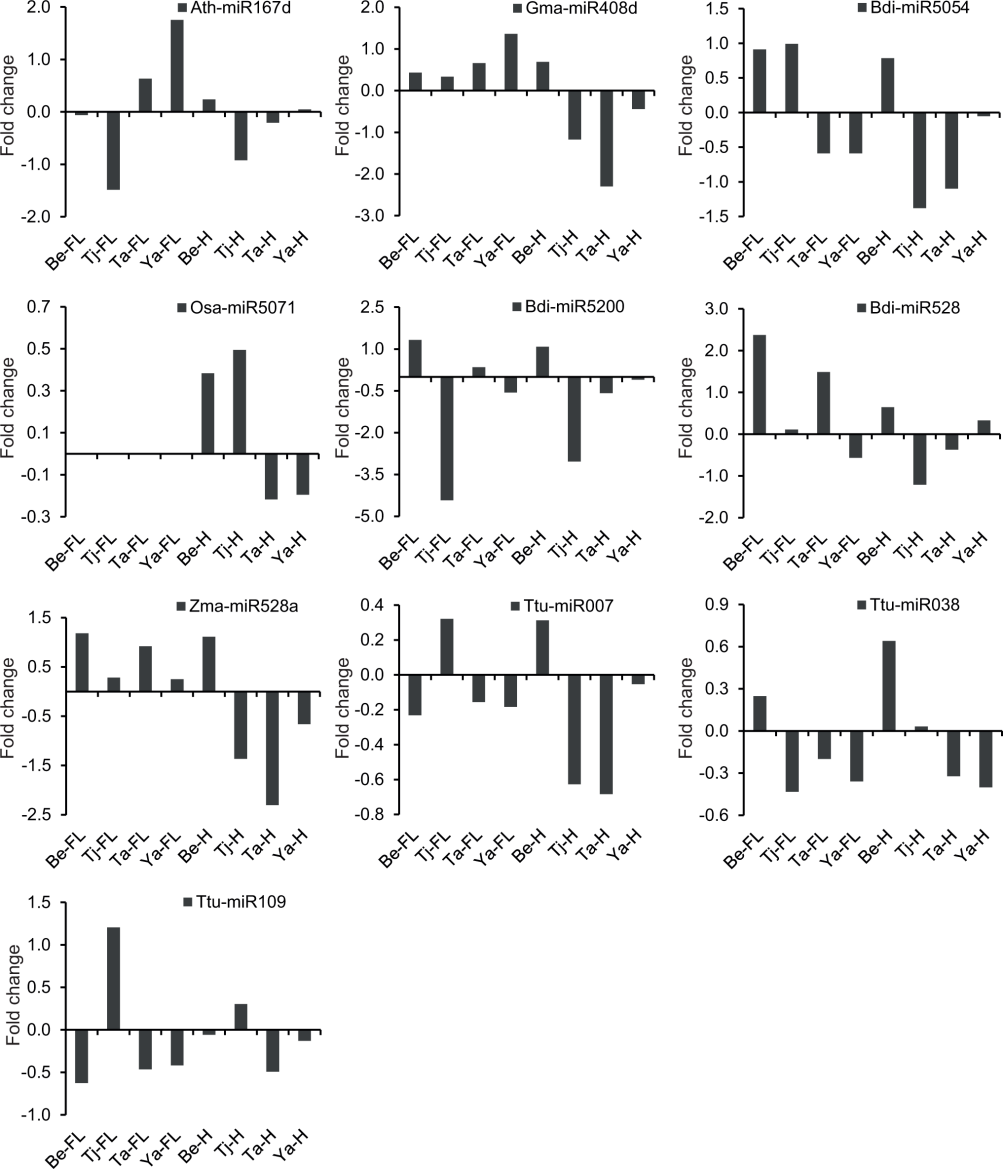
Expression analysis of stress responsive miRNA candidates by qPCR in four durum wheat genotypes. GAPDH was used as an endogenous control. The fold change is shown as a log2 value of miRNA expression in the water deficit libraries/miRNA expression in the control libraries. FL = Flag leaf samples; H = Head samples; Be = EGA Bellaroi; Ta = Tamaroi; Tj = Tjilkuri; Ya = Yawa; Ath = *Arabidopsis thaliana*; Bdi = *Brachypodium distachyon*; Gma = *Glycine max*; Osa = *Oryza sativa*; Ttu = *Triticum turgidum*; Zma = *Zea mays*.

To validate differentially expressed durum miRNAs predicted by high-throughput sequencing, miRNA was quantified using qPCR. Ten selected stress responsive durum miRNA candidates including seven conserved miRNAs (identical to Ath-miR167d, Gma-miR408d, Bdi-miR5054, Osa-miR5071, Bdi-miR5200, Bdi-miR528 and Zma-miR528a) and three novel miRNAs (Ttu-miR007, Ttu-miR038 and Ttu-miR109) were screened using flag leaf and developing head tissues of four durum genotypes simultaneously. Comparative fold changes of expression levels of miRNA are shown in Fig 7. The expression level changes of conserved miRNAs detected by qPCR were compared with those determined by Illumina sequencing (S5 Table). Most miRNAs showed similar trends in their expression profile across Illumina sequencing results and qPCR results. For example, in the Illumina sequencing analysis, Zma-miR528a was determined to be down-regulated under stress in the head libraries of Tjilkuri, Tamaroi and Yawa (1.2, 5.1, and 2.7 fold reduction), and up-regulated in EGA Bellaroi (3.6 fold increase). When tested by qPCR, the same miRNA was up/down-regulated in the same libraries and varieties (2.6, 4.9, 1.6 fold reduction, and 2.2 fold increase, respectively). While the expression values between the two platforms are not exactly the same, this has been reported previously and is expected based on the two different quantification methods used [47].

### Putative targets of novel water deficit stress responsive durum miRNAs, GO analysis and qPCR

To infer the biological functions of the novel water deficit stress responsive miRNAs in durum, putative mRNA target genes were predicted using the psRNAtarget program (http://plantgrn.noble.org/psRNATarget/) with the wheat DFCI gene index (TAGI) version 12 as a reference. A total of 130 targets were identified for four novel stress responsive durum miRNAs (S6 Table). Ttu-miR008 had the highest number of putative target genes (101) while Ttu-miR109 had the lowest (5). On the basis of sequence complementarity between miRNAs and putative target genes, the possible inhibition type between miRNA and their targets was predicted [48,49]. Out of 130 predicted mRNA targets, the inhibition of 109 mRNA targets (83.8%) is caused by cleavage activity, while 21 targets (16.2%) are inhibited through translational repression (S6 Table).

**Fig 8:**
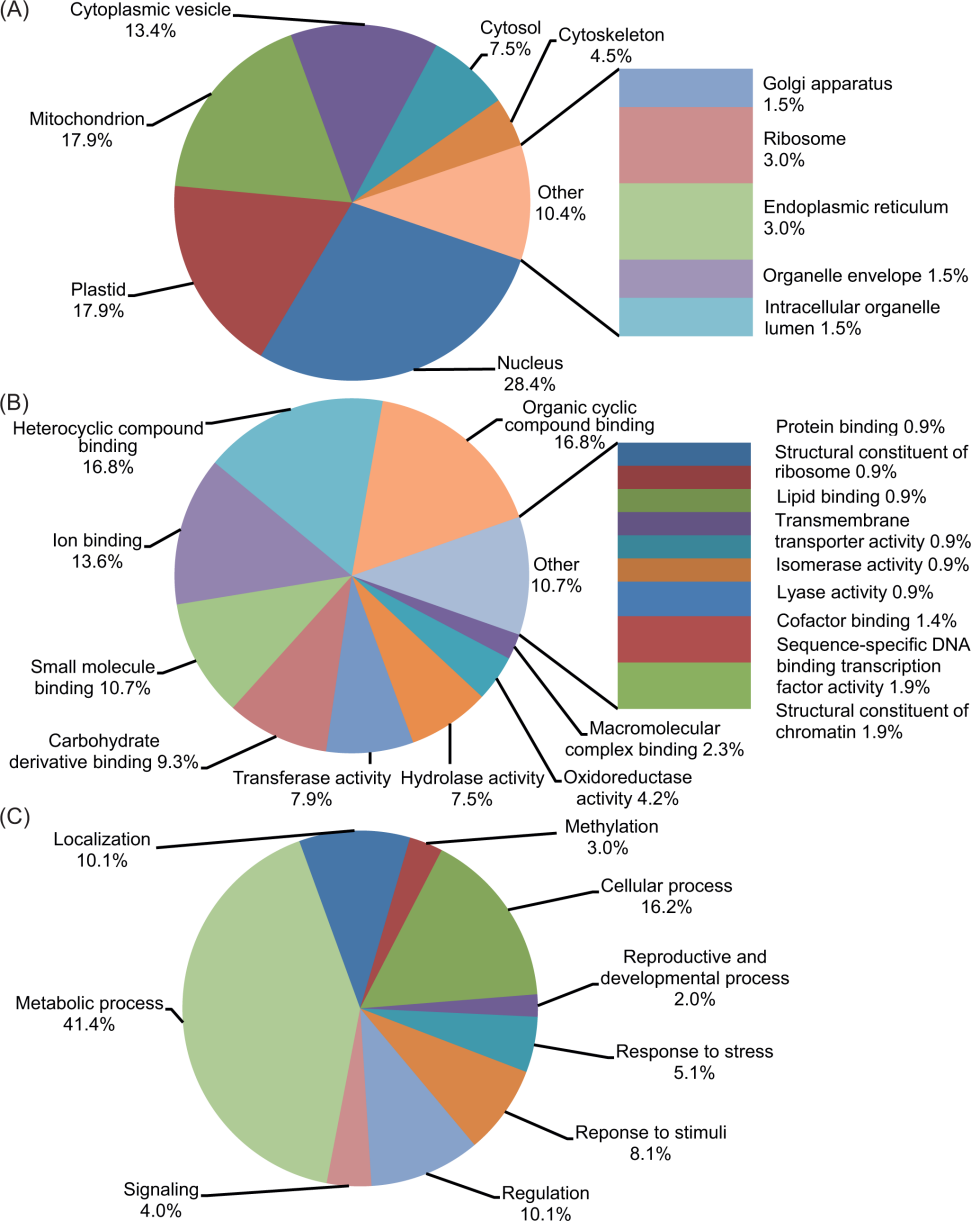
Categorisation of predicted targets of four novel stress responsive miRNAs using Gene Ontology (GO) terms. Pie charts representing different GO categories are based on the number of target sequences enriched in each GO term. GO terms at level 8 are used for (A) cellular component categorisation. GO terms at level 3 are used in categorisation for (B) Molecular function, and (C) Biological processes. The percentage of each GO term is based on the number of targets enriched for that term relative to the total number of targets in each category. The GO level represents the position of a GO term in the GO hierarchy. The level of a GO term is the number of GO terms between that term and the Root Term of the Ontology.

All of the predicted targets were analysed through Gene Ontology (GO) using the Blast2GO server (https://www.blast2go.com/) to further evaluate their putative functions (S6 Table). The BLASTX search obtained the most significant BLAST hits for each target across different species (S3 Fig). According to the ontological definitions of their GO terms, all targets were grouped into three GO categories (S7 Table). At the cellular level (Fig 8A), predicted targets are primarily associated with the nucleus (28.4%), followed by either the mitochondrion or plastid (17.9% each). In evaluating molecular functions, the majority of the targets are potentially involved in either organic or heterocyclic compound binding (16.8% each), ion binding (13.6%), or small molecule binding (10.7%) (Fig 8B). Biologically, nearly half of the targets were classified as being involved in metabolic processes (41.4%) (which includes catabolic, cellular, nitrogen compound, organic substance, primary, and wax metabolic processes) (Fig 8C). The remaining targets were involved in a broad range of biological processes including cellular processes (16.2%), regulation (10.1%), localisation (10.1%), response to stimuli (8.1%), and most significantly, response to stress (5.1%) (Fig 8C). Many of the predicted targets are annotated to be transcription factors, elongation factors, protein phosphatases, and osmotic stress receptors that are associated with multiple stress response processes (S6 Table).

Seven selected targets of Ttu-miR008 were quantified using qPCR (S8 Table). For example, TC438017 (non-specific lipid-transfer protein) and CV779294 (non-specific lipid-transfer protein a-like). In the flag leaf under water stress, TC438017 was up-regulated in the stress tolerant genotypes (4.26 fold in Tamaroi and 2.79 fold in Yawa), whereas it was down-regulated in the stress sensitive genotypes (2.72 fold in EGA Bellaroi and 1.11 fold in Tjilkuri). Similarly, CV779294 was up-regulated in the stress tolerant genotypes (1.34 fold in Tamaroi and 1.40 fold in Yawa), while being down-regulated in the sensitive genotypes (2.37 fold in EGA Bellaroi and 2.41 fold in Tjilkuri). In addition, TC447684 (Glossy 1 protein–GL1) was shown to be up-regulated in the developing head of the stress tolerant genotypes (1.22 fold in Tamaroi and 1.13 fold in Yawa), while being down-regulated in the developing head of the sensitive genotypes (1.17 fold in EGA Bellaroi and 1.52 fold in Tjilkuri). Overall, of the seven targets quantified several were negatively correlated with Ttu-miR008, which was down-regulated in the stress tolerant genotypes but up-regulated in the stress sensitive genotypes (Fig 6B).

## Discussion

### The miRNAome in durum wheat under water deficit stress

Water deficit is a major abiotic stress that limits the production of many crops in rain-fed environments. Plant responses to water deficit stress are regulated by complex genetic and epigenetic networks. Interactions between miRNAs and their target mRNAs through sequence-specific binding offer an inheritable and accurate regulation pathway for plants to respond to environmental stimuli at both the translational and post-transcriptional level. To date, extensive efforts have been made to discover water deficit stress-associated miRNAs in many plants including Arabidopsis [24], rice [22], maize [50], soybean [51], barley [52] and bread wheat [16,53]. However, there has rarely been any study on water deficit-stress responsive miRNAs in *Triticum turgidum*, with only the ssp. *dicoccoides* being investigated but under shock drought conditions [23]. As an important cereal, mostly grown in rain-fed Mediterranean environments under stressful and variable conditions, durum wheat offers an attractive alternative to studying the much more complex bread wheat genome. With climate change models predicting increased rising temperatures and decreased rainfall, understanding the water deficit stress response pathway(s) in durum wheat has become an important research objective for breeding programs.

Using deep sequencing of small RNA libraries in this study, we discovered significant changes that occur with the miRNAome in four durum genotypes under water deficit stress and across two tissue types. Illumina sequencing yielded approximately 623 million reads which were subsequently trimmed and processed to remove inherent redundancy, obtaining a total of 301 million unique sRNA sequences. The highest proportion of the sequenced RNAs was 24 nt in length, which is in agreement with previous studies where 24 nt sRNA fragments constituted the majority of small RNA populations, thereby implicating the function of Dicer proteins during the formation of miRNAs [25,29,54]. Since durum wheat (2*n* = 4*x* = 28, genomes AABB) is an ancestral source of the A and B genomes of bread wheat (2*n* = 6*x* = 42, genomes AABBDD) and only a partial genome sequence for *Triticum turgidum* ssp. *durum* is available, the International Wheat Genome Sequencing Consortium’s (IWGSC) Chromosomal Survey Sequences (CSS) of bread wheat was used to identify novel putative miRNA hairpins in durum sRNA libraries [44]. From the 110 conserved miRNAs and 159 novel miRNA hairpins identified, 66 conserved miRNAs and four novel miRNAs were water deficit stress responsive. Further experimental validation including Poly (A)-qPCR and miRNA* examination will assist in confirming novel durum miRNA hairpins and their precise excision of the miRNA/miRNA* duplex [20,55]. In this study, ten representative stress responsive miRNAs (seven conserved and three novel) were validated by Poly (A)-qPCR. Poly (A)-qPCR has been shown in bread wheat to provide more accurate and consistent quantification of miRNA expression than stem-loop qPCR [56].

Among water deficit stress responsive miRNAs identified in this study, some miRNAs have been found to be associated with abiotic stress response in previous studies; including miR156, miR159, miR167, miR319, miR393, miR398, and miR408. The expression patterns of some of these water deficit stress responsive miRNA were similar to results previously reported. For example, miR159 was up-regulated 1.75 times under water deficit stress in Tjilkuri. Similarly in maize, the expression level of miR159 was significantly increased during drought stress [21]. The up-regulation of miR162, miR167, miR393 under water deficit stress has been commonly observed in different plants (Table 1), indicating that some miRNA stress-responsive pathways are more than likely to be conserved across different plant species including durum wheat. In contrast, some conserved miRNAs, as well as novel durum miRNAs, were found to be water deficit stress responsive for the first time, including miR1136, miR1432, miR5048, miR5054, miR5071, miR5200 and miR6300. Their regulation pattern indicates that these miRNAs are possibly involved in species-specific response pathways.

Most interestingly, the expression profiles of 16 conserved and five novel miRNA hairpins showed inverted regulatory patterns between water deficit stress tolerant and sensitive genotypes, suggesting the regulatory roles of miRNAs in some stress response pathways are genotype-specific (Fig 6). The four durum wheat genotypes used in this study have different levels of water deficit tolerance, which is reflected through their genotypic physiological responses [1]. The distinct genotype differences in miRNA expression profiles could lead to inverted regulation of their functional target genes, which might activate different physiological responses between genotypes [16]. In a recent study of dehydration associated miRNA in wheat, contrasting expression patterns of 13 conserved miRNA (including Tae-miR160a, Tae-miR166h, Tae-miR172a, and Tae-miR393) were also observed between stress tolerant and sensitive genotypes [16]. In the current study, several conserved miRNAs were found to be predominantly expressed in specific genotypes, with or without water deficit stress treatments. For example, miR5200 was consistently more abundant in the water deficit stress sensitive genotypes (EGA Bellaroi and Tjilkuri) than the stress tolerant genotypes (Tamaroi and Yawa) in both the control and water deficit stress libraries. Based on the prediction and further analysis of miRNA targets, we can infer that different capacities for water deficit stress tolerance between durum wheat genotypes may arise from the differential physiological regulation triggered by target genes, which are regulated by genotypic stress responsive miRNAs.

### Regulation of miRNA and their targets may contribute to genotypic variation in stress tolerance capacity in different durum genotypes

In the present study, *in silico* target gene predictions and GO analysis were carried out for four novel water deficit stress responsive miRNAs. This bioinformatics strategy has been applied previously in bread wheat to successfully predict and construct possible miRNA/mRNA target stress regulatory pathways, which were further experimentally validated [16,19,53,57–59]. A total of 130 target genes for four novel durum miRNAs were predicted to encode proteins of diverse functions. GO analysis indicated that these targets are involved in a broad range of biological processes and varied physiological responses in durum wheat, such as biosynthetic activity, binding activities with proteins and nucleic acids, protein transport, abscisic acid (ABA) metabolic processes, photosynthetic activity and leaf senescence. Significantly, stress responsive expression of seven predicted target genes were validated by qPCR. The negative correlation of several targets with their corresponding miRNA implies the involvement of miRNA-mRNA target regulation in stress response pathways in durum.

A significant number of targets are predicted to possess nucleic acid binding activities and encode transcription factors involved in signalling and defence, which contribute to stress tolerance in different durum genotypes. For example, auxin response factor (ARF) 18-like is a target of Ttu-miR008. ARFs bind to auxin response elements to usually negatively regulate expression of auxin-inducible genes such as *GH3* (Gretchen Hagen3), *Aux*/*IAA* (auxin/indole-3-acetic acid) and *SAUR* (small auxin-up RNA) [60]. Several auxin-responsive genes have been identified to respond to various abiotic stress conditions such as drought, salinity and cold in Arabidopsis, rice and sorghum, indicating the cross-talk between auxin signalling and abiotic stress responses [61–63]. In durum, Ttu-miR008 is down-regulated under stress in the tolerant genotypes suggesting that ARF18-like protein increases thereby repressing auxin-inducible genes enhancing auxin signalling. This might affect processes which require a lower auxin:cytokinin ratio, such as lateral root development [64]. In maize and wheat, the development of lateral roots in the stress tolerant genotype is enhanced from the accumulation of auxin-responsive factors [16,17]. However, the role of miRNA and ARF in lateral root development in durum needs to be confirmed with further experimental validation.

Other targets also contribute to water stress tolerance in durum as signalling factors including protein kinases and protein phosphatases. For example, a target of Ttu-miR008 (TC451175) was annotated as a probable protein phosphatase 2C (PP2C). Studies in Arabidopsis and rice have shown that PP2C genes were induced by diverse environmental stimuli and acted as positive regulators in ABA-mediated signalling pathways well known to be involved in stress responses [65,66].

However, there are also other targets of Ttu-miR008 which could contribute to water deficit stress tolerance in different ways such as maintaining osmotic pressure of the plant or homeostasis of the cell. For example, the target CV769573 identified in this study as an ABA 8'-hydroxylase, is a key enzyme in ABA degradation [67]. ABA is crucial for various stress responses, including regulation of stress-responsive genes, stomatal closure, and metabolic changes [68]. ABA is rapidly increased in response to environmental stress [67], suggesting a role for removing ABA 8'-hydroxylation to ensure increased ABA levels. Equally rapid elimination of stress induced ABA when stresses are relieved is essential [69]. Indeed, dehydration stress can cause steady increases in ABA degradation in Arabidopsis over time [70]. Although requiring confirmation, ABA 8'-hydroxylase may therefore decrease to a lesser extent in tolerant genotypes suggesting they have a lower ABA requirement during water deficit stress.

Also identified and quantified in this study was the Glossy 1 (GL1) protein, which is yet another target of Ttu-miR008 (TC447684). GL1 functions in the biosynthesis pathway of cuticular wax, which provides protection against environmental stress. In rice, Os*GL1* over-expression plants showed increased cuticular wax accumulation on the leaf surface and were more tolerant to drought stress at reproductive stages compared to the wild type [71]. The inhibition of GL1 is reduced through the down-regulation of stress responsive miRNA, leading to enhanced wax production, thus preventing water loss. This helps to explain the genotypic difference in the reduction of relative water content in leaves, in response to water deficit stress between stress tolerant and sensitive durum genotypes [1].

Two other quantified functional targets, TC438017 (non-specific lipid-transfer protein) and CV779294 (non-specific lipid-transfer protein a-like), examined by qPCR may also assist to explain the genotypic difference in maintaining osmotic pressure. Lipid transfer proteins (LTPs) help to repair stress-induced damage in membranes or alter the lipid composition of membranes. In pepper, the accumulation of LTP transcripts induced by environmental stresses is associated with cuticle formation, which contributes to the avoidance/tolerance of low tissue water potential and water content [72,73]. In this study, TC438017 and CV779294 were negatively correlated with their corresponding miRNA showing genotypic expression patterns in response to water deficit. The up-regulated accumulation of LTPs observed only in stress tolerant durum genotypes helps to explain the genotypic differences in the maintenance of leaf water potential and relative water content [1], suggesting the participation of miRNA/target interaction in genotypic physiological response pathways in durum. Experimental examination of these miRNA-regulated targets also helps demonstrate the validity of prediction analysis using bioinformatics.

## Conclusion

The present study provides a comparative description of the miRNAome in durum wheat between water deficit tolerant and sensitive genotypes in response to water deficit stress, suggesting that there are multiple miRNA regulation patterns which might contribute to, and partly explain, the distinct water deficit stress sensitivities between different durum genotypes. The first comprehensive durum small RNA dataset generated provides a good foundation for future characterisation of the molecular mechanisms underlying water deficit stress tolerance in durum. This was achieved through Illumina sequencing, which enabled profiling of the miRNAome in water deficit stress tolerant and sensitive durum wheat genotypes across different tissues and treatments. We have identified 110 conserved miRNAs and 159 novel miRNA hairpins in durum wheat, including 66 conserved miRNAs and five novel miRNA hairpins (representing four novel miRNAs) that are water deficit stress responsive. A total of 16 conserved miRNA hairpins (representing 11 conserved miRNAs) and five novel miRNA hairpins (representing four novel miRNAs) showed distinct down-regulation profiles in the water deficit stress tolerant genotypes while the same miRNAs were up-regulated in sensitive genotypes. This demonstrates that regulation patterns of the same miRNAs may vary extensively across genotypes of the same species, in response to environmental stimuli. Target prediction and GO analysis of four novel genotype-specific regulated miRNAs provide evidence for the potential involvement of miRNAs in a broad range of biological processes, including stress response pathways. Several potentially valuable target genes have been identified and are now undergoing further experimental validation, which will be reported elsewhere.

## Materials and Methods

### Plant material and growth conditions

Four durum wheat genotypes (EGA Bellaroi, Tamaroi, Tjilkuri and Yawa) were used in this study. Seeds were obtained from Durum Breeding Australia’s (DBA) southern node breeding program (The University of Adelaide). Tamaroi and Yawa are water deficit stress tolerant genotypes; while EGA Bellaroi and Tjilkuri are water deficit stress sensitive genotypes [1]. Plants were grown at 22°C/12°C day/night temperature with a 12 h photoperiod with watering to field capacity (12% soil water content (SWC)) from germination to booting stage when the water limiting stress treatment was imposed for 15 d (6% SWC or 50% field capacity; water deficit stress group, WG) or field capacity maintained (control, CG), as per Liu et al. [1].

### Sampling and total RNA extraction

After 15 d of water deficit stress, the flag leaf and developing head were collected with sterile razor blades and frozen immediately in liquid nitrogen. Frozen tissues were ground to a fine powder in liquid nitrogen using a sterile mortar and pestle, pre-chilled to -80°C. Total RNA was isolated using the TriPure isolation reagent kit (Roche Diagnostics, Australia) and treated with RQ1 RNase-Free DNase I (Promega, Australia) following the manufacturer’s instructions. The concentration and quality of extracted RNA samples were measured by spectrophotometric analysis at 260 nm and 280 nm using a NanoDrop Lite spectrophotometer (Thermo Scientific, USA). RNA integrity was assessed by agarose gel electrophoresis. A total of 96 RNA samples (4 durum genotypes × 2 tissue types × 2 treatment groups × 6 biological replicates = 96) were extracted and stored at -80°C for downstream applications.

### Small RNA library construction and deep sequencing

For small RNA library construction, 5 μg of total RNA was size-fractionated on a 15% denaturing TBE urea polyacrylamide gel and small RNAs (15 to 40 nt) were excised using an NEB miRNA marker (New England Biolabs, UK) as a guide. Small RNAs was eluted in 0.3 M NaCl by rotating the tube overnight at 4°C. Eluted RNA was passed through a Spin-X column and then precipitated using glycoblue (Ambion, USA) and isopropanol. The sRNA pellets were washed and air-dried at room temperature, then re-suspended in DEPC-treated water [74]. A total of 96 small RNA libraries were constructed from flag leaf and developing head of durum wheat plants that had been treated or not treated with water deficit stress (4 durum genotypes × 2 tissue types × 2 treatment groups × 6 biological replicates = 96) using NEB Next^®^ Multiplex Small RNA Library Prep Set for Illumina (New England Biolabs, UK) following the manufacturer’s instructions. For each flag leaf sRNA library and head sRNA library, a unique index primer was used for multiplexing purposes using the NEBNext^®^ Index Primer Set (New England Biolabs, UK). The final cDNA product was purified using Pippin Prep™ System (Sage Science, USA). Prior to sequencing, quality and quantity of the amplified small RNA cDNA libraries was evaluated on an Agilent 2100 Bioanalyzer system (Agilent Technologies, USA) and Qubit fluorometer (Invitrogen, USA). All 96 small RNA libraries were sequenced using Illumina sequencing technology on a HiSeq2500 machine after cluster generation. All sequencing reads were submitted to the NCBI GEO database (http://www.ncbi.nlm.nih.gov/geo/), and are accessible under the accession number GSE69339.

### Identification of conserved miRNAs (Approach #1)

In this study, Approach #1 was developed to identify conserved miRNAs in durum wheat using CLC Genomics Workbench v7.0 (CLC Bio, Denmark). Briefly, raw sequencing reads were first processed by trimming adaptor sequences and removing low-quality reads. Sequences shorter than 15 nt and larger than 50 nt were excluded from further analysis. Trimmed reads were generated for each small RNA library and then annotated to determine the presence of known plant miRNAs. Durum small RNA sequences were aligned with known miRNAs in miRBase using CLC Genomics Workbench v7.0 based on their sequence homology, allowing up to two mismatches in alignment [15]. Conserved miRNAs in common monocot and dicot species (*Triticum aestivum*, *Triticum turgidum*, *Brachypodium distachyon*, *Zea mays*, *Oryza sativa*, *Hordeum vulgare*, *Sorghum bicolor*, *Arabidopsis thaliana*, and *Glycine max*) deposited at miRBase v20 (June 2013) were used as references for annotation. Normalisation of miRNA abundance in each library was carried out using a value referred to as RPM (reads per million). The RPM value was obtained by dividing the reads number of a miRNA with the total number of putative sRNA reads in each library and multiplying by a million. Matched sequences with no more than two mismatches and with an abundance of over two RPM in at least 50% of the 96 libraries were considered as candidate conserved miRNAs.

### Identification of differentially expressed conserved miRNAs (Approach #1)

Differentially expressed conserved miRNAs were identified based on the RPM. To identify differentially expressed miRNAs, the following criteria were used: 1) number of miRNA reads was set as 0.01 by default when the sequencing read was 0; 2) normalised reads (RPM) was at least 10 in one of the libraries in comparison; and 3) the fold-change of normalised reads of libraries in comparison was greater than 1.5 [16,75]. For expression analysis, reads of unique mature miRNAs deposited in miRBase were used as they are an active and functional form of mature miRNAs [29]. Tissue-specific conserved miRNAs were identified by comparing flag leaf libraries with head libraries. Genotype-specific conserved miRNAs were identified by comparing water deficit sensitive varieties and water deficit tolerant varieties. Comparisons were made only between EGA Bellaroi and Tamaroi, or Tjilkuri and Yawa due to their breeding background. Water deficit stress-responsive miRNAs were identified by comparing control treatment libraries with water deficit stress treatment libraries. Heat maps of differentially expressed miRNAs were generated in R (version 3.1.2) (http://www.r-project.org/). Where the fold change of some conserved miRNA candidates were not analysed due to their low reads in the sequencing results (RPM were less than 10 in both libraries for differential expression comparison), their log2 fold change under stress was recorded as zero in the clustering analysis.

### Small RNA-Seq data pre-processing for novel miRNA identification (Approach #2)

To identify novel miRNAs in durum wheat, a customised bioinformatics approach (Approach #2) was developed. Small RNA-Seq raw reads were 5' and 3' adapter trimmed and the output partitioned into two sets of reads: 1) those that had been trimmed and were 19–26 bp long, and 2) those that did not contain any adapter sequence. The first set represents non-redundant (NR) 3' adapter trimmed reads, which were used to identify putative pre-miRNA hairpin. In order to remove reads which are derived from the breakdown products of longer mRNA's rather than true sRNA molecules, the second set of reads and the NR sRNA reads were *de novo* assembled to generate a reference against which sRNA reads would be filtered. This was done using Velvet (v 1.2.09, https://www.ebi.ac.uk/~zerbino/velvet/) with a kmer length of 17 and read tracking enabled. The NR set of 3' adapter trimmed reads (19–26 bp) were filtered to remove those which were either: a) low abundance (< = 5 reads in all samples); b) mapped to known wheat rRNAs; c) mapped to the wheat chloroplast or mitochondrial genomes; d) mapped to the 50bp+ long *de novo* assembled contigs; e) mapped to UniVec (build 7.1, http://www.ncbi.nlm.nih.gov/VecScreen/UniVec.html) data set; or f) mapped to the Triticeae Repeat Sequence Database (TREP) database of grass repeat sequences [76]. The mappings in steps b-f above was performed using Bowtie2 (v 2.2.3; http://bowtie-bio.sourceforge.net/bowtie2/index.shtml) with parameters which allowed up to two mismatches and no indels. The NR reads which passed all the above filters were used to identify candidate pre-miRNA hairpins.

### Identification of miRNA precursors and novel miRNA candidates in durum wheat (Approach #2)

Since only a partial genome sequence for *Triticum turgidum* ssp. *durum* is available, the International Wheat Genome Sequencing Consortium’s (IWGSC) Chromosomal Survey Sequences (CSS) [44] was used to identify putative miRNAs. The NR sRNA sequences which passed the filters were mapped to the IWGSC CSS using BioKanga v3.4.3 (http://sourceforge.net/projects/biokanga/) in order to identify all possible contigs from which the sRNA sequence could have been derived. For each NR 3' adapter trimmed read, all perfect alignment locations in the IWGSC CSS were identified. Using a subset of reads and CSS contigs involved in those perfect alignments, we also identified all imperfect alignments (two-five mismatches). The candidate pre-miRNA hairpins were defined using all pairwise combinations of perfect to imperfect alignments of a given read within a CSS contig. Additional constraints were applied such that the perfect and imperfect alignments were in opposite orientations and separated by 54–1000 bp. A NR set of these regions ±20 bp, were processed by RNAFold (http://rna.tbi.univie.ac.at/cgi-bin/RNAfold.cgi) and then miRcheck (http://web.wi.mit.edu/bartel/pub/software.html) to ascertain if they could form hairpin structures with characteristics associated with the miRNA biogenesis pathway in plants, indicating the formation of a miRNA/miRNA* duplex from stem-loop hairpins based on their read coverage profile [55]. Three primary criteria were applied as follows: 1) A peak of reads in the first or last 50 bp of the hairpin sequence all aligned to the same strand/stem (the miRNA site); 2) a second, smaller peak of complementary reads aligned on the opposite end to the miRNA strand/stem (the miRNA* site); 3) a small proportion of reads mapping between the above two defined regions (the loop). All candidate miRNA hairpin sequences were classified into one of eight categories (A-H, where A has a read coverage profile matching the expectations for a true miRNA) using three Boolean metrics based on their read coverage profile: 1) if ≥95% of the reads mapped to one strand of the hairpin; 2) if ≥95% of the reads mapped to one of the terminal 50 bp of the hairpin; and; 3) if ≤5% of the reads mapped to the loop region of the hairpin (Table 2). Putative miRNA hairpins were further characterised by identifying if their sequence contained any perfect matches to the 35,828 mature miRNAs from miRBase v21 (accessed July 2014).

### Identification of stress responsive novel miRNA hairpins (Approach #2)

To identify novel water deficit stress-responsive miRNA hairpins, the Limma Bioconductor (v3.18.13) package [45] was used to perform a statistical analysis using linear models based on the RPM data. Different durum varieties were recoded with binary values which indicated water deficit stress tolerance or water deficit stress sensitivity. Of the many possible contrasts that could be made, the tolerance × treatment interaction term was of primary interest in the linear model. This effectively identified hairpins which showed differential expression to water deficit stress and that this response was different between water deficit stress sensitive and water deficit stress tolerant cultivars. Pre-miRNA hairpins from Category A (Table 2) which had a significant tolerance × treatment interaction were then inspected to ascertain if their read-coverage profiles followed what we expected from a true mature miRNA and miRNA*.

### Quantitative real-time PCR (qPCR) of miRNA candidates

In order to evaluate the expression of miRNA candidates, poly-A tailing combined with qPCR was performed for a select group of seven conserved and three novel stress responsive miRNAs with the 96 durum total RNA samples which were used for sRNA library construction. For each sample, 1 μg of total RNA was poly-A tailed and reverse-transcribed with the NCode VILO miRNA cDNA synthesis kit (Invitrogen, USA) following the manufacturer’s instructions. The final cDNA product was diluted to 100 μL. qPCR was performed using the ViiA™ 7 Real-Time PCR system (Applied Biosystems, USA). In each 10 μL qPCR reaction (six biological replicates for each sample), 1 μL diluted cDNA template and primers (3 pmol of each forward and reverse) were mixed with SYBR^®^ Green reagent (iQ ^TM^ supermix, BioRad, USA). The forward miRNA primers were designed based on the full mature miRNA sequences (S9 Table). The reverse primer was the universal reverse primer provided in the NCode VILO miRNA cDNA synthesis kit. The qPCR running conditions were: 95°C for 2 min, followed by 40 cycles of 95°C for 15 s, 56/58/60°C for 15 s, and 70°C for 10 s, followed by 72°C for 10 min. Melting curve analysis was used to detect the specificity of the amplified product. The relative expression ratio was calculated using the comparative CT (^ΔΔ^ C_T_) method with GAPDH [GenBank: AF251217] as the reference gene.

### Target prediction, functional GO analysis and target qPCR

The putative mRNA targets of stress responsive novel miRNAs were identified using psRNA Target Server (http://plantgrn.noble.org/psRNATarget/) with the following parameters: prediction score cut-off value = 3.0, length for complementarity scoring = 20, and target accessibility = 25. Mature novel miRNA sequences were used as queries and the wheat DFCI gene index (TAGI) version 12 was used as the reference genome dataset [19]. All the predicted targets were evaluated using the functional enrichment analysis tool at Blast2GO (http://www.blast2go.com) [77,78]. BLASTX was employed to perform a homology search against the NR protein databases in NCBI to obtain the most significant BLAST hits for each target using the Blast function with Blast2GO. Default parameters were used in the mapping and annotation steps to obtain GO terms for each target transcript in Blast2GO. The annotation results were further improved by analysing conserved domains/families using the InterProScan function. GO terms for three categories (cellular component, molecular function and biological processes) were determined for each annotated target. All the annotated targets were classified on the basis of their GO term enrichments in each category. Seven selected functional targets were quantified using qPCR with the same cDNA libraries employed in the miRNA qPCR. Target qPCR was performed using the comparative CT (^ΔΔ^ C_T_) method with GAPDH as the reference gene [GenBank: AF251217]. Target primers were designed to include the predicted miRNA/mRNA binding region in the amplified product ensuring the quantification of uncleaved targets, in order to examine the correlation of miRNA and regulated targets. Target transcript sequences, primer locations and primer sequences are listed in S10 Table.

## Supporting Information

S1 Fig The length distribution of small RNA reads obtained by high-throughput sequencing in durum wheat. Only one representative library (from a total of 96 libraries) is shown. All sequencing reads were submitted to the NCBI GEO database (http://www.ncbi.nlm.nih.gov/geo/), and are accessible under the accession number GSE69339.
(EPS)

S2 Fig Predicted secondary structures of five novel durum miRNA hairpins that are responsive to water deficit stress. Mature miRNAs are highlighted in blue while miRNA* are highlighted yellow. The secondary structures of the novel durum wheat miRNA hairpins (A) Ttu-pre-miR007, (B) Ttu-pre-miR008, (C) Ttu-pre-miR038, (D) Ttu-pre-miR109, and (E) Ttu-pre-miR119 are shown.
(TIF)

S3 Fig Species distribution of all BLAST hit alignments from the GO analysis. Identified target gene transcripts are searched in the species-specific entries registered in the GO database. Species distribution is based on the number of BLAST hits aligned in each species.
(TIF)

S1 Table Sequencing reads and the output data obtained from the CLC Genomics workbench pipeline. Data is shown for 96 libraries presented in 16 different biological library pools (four genotypes × two tissue types × two treatments). CG = Control group; WG = Water deficit stress group; FL = Flag leaf libraries; H = Head libraries.
(XLS)

S2 Table List of known microRNAs in durum wheat and their normalised reads in each library. CG = Control group; WG = Water deficit stress group; FL = Flag leaf libraries; H = Head libraries.
(XLS)

S3 Table List of novel durum microRNA hairpins and their normalised reads in each library. CG = Control group; WG = Water deficit stress group; FL = Flag leaf libraries; H = Head libraries. The Hairpin Alignment Identifier is derived from the genome location information of the hairpin sequence in the IWGSC CSS (International Wheat Genome Sequencing Consortium’s Chromosomal Survey Sequences), as well as the alignment position and the length of the reads used to identify putative microRNA hairpins. The Hairpin Alignment Identifiers take the following form as an example: 1AL_3896362:3010–3120[21,21]. 1AL_3896362 = the sequence identifier in the IWGSC CSS, this is from chromosomal arm 1AL; 3010 = position of the first base of the hairpin within the IWGSC CSS; 3120 = position of the last base of the hairpin within the IWGSC CSS; [21,21] = the putative mature miRNA starts at position 21 in the hairpin and is 21 bp in length.
(XLS)

S4 Table Conserved water deficit stress responsive miRNAs in durum wheat. Fold changes have been determined by comparing the RPM between the control treatment libraries and the water deficit stress treatment libraries in the flag leaf and the developing head of four durum wheat genotypes. Fold changes are shown when greater than 1.5 fold. Green values indicate that miRNA reads were more abundant in the water deficit treatment libraries. Red values indicate that the miRNA reads were more abundant in the control treatment libraries. CG = Control group; WG = Water deficit stress group; FL = Flag leaf libraries; H = Head libraries; Be = EGA Bellaroi; Ta = Tamaroi; Tj = Tjilkuri; Ya = Yawa; Ath = *Arabidopsis thaliana*; Bdi = *Brachypodium distachyon*; Gma = *Glycine max*; Hvu = *Hordeum vulgare*; Osa = *Oryza sativa*; Sbi = *Sorghum bicolor*; Tae = *Triticum aestivum*; Zma = *Zea mays*.
(XLS)

S5 Table Fold-change of selected water deficit stress responsive miRNA candidates identified by Illumina sequencing and qPCR. Fold changes have been determined by comparing the RPM in Illumina sequencing or comparing relative expression ratio in qPCR between the control treatment and the water deficit stress treatment in different tissues of four durum wheat genotypes. Bold fold change value indicates that the miRNA was more abundant in the water deficit stress treatment libraries whereas unbolded fold change indicates that the miRNA was more abundant in the control treatment libraries. FL = Flag leaf libraries; H = Head libraries; CG = Control group; WG = Water deficit stress group; Be = EGA Bellaroi; Ta = Tamaroi; Tj = Tjilkuri; Ya = Yawa; Ath = *Arabidopsis thaliana*; Bdi = *Brachypodium distachyon*; Gma = *Glycine max*; Osa = *Oryza sativa*; Zma = *Zea mays*.
(XLS)

S6 Table Predicted targets of four novel durum stress responsive miRNAs and their GO analysis results. Definitions: Column E (Expectation)–The expectation scoring of the complementarity between miRNAs and their targets. The maximum expectation threshold score was set at 3.0. Column F (Target Accessibility (UPE))–The maximum energy required to open (unpair) the secondary structure around the target site on the target mRNA. Column O (Multiplicity)–Multiplicity of the target site representing the number of target sites within a specific target transcript.
(XLS)

S7 Table Combined Gene Ontology classification in GO levels of 130 predicted targets of four novel miRNAs. Definitions: Column A (Level)–The GO level represents the position of a GO term in the GO hierarchy. The level of a GO term is the number of GO terms between that term and the Root Term of the Ontology. Column E (Node score)–The node score is the sum of sequences directly or indirectly associated to a given GO term weighted by the distance of this term to the term of its direct annotation, i.e. the GO term the sequence is originally annotated to. This confluence score takes into account the number of sequences converging at one GO term and at the same time penalises by the distance to the term where each sequence was actually annotated. Column F (%Seq)–The percentage of sequences annotated with a particular GO term among all the sequences annotated within the same GO level. Column G (#Seq)–The number of target sequences annotated with that particular GO term.
(XLS)

S8 Table Fold-change of seven selected functional targets of Ttu-miR008 quantified by qPCR. Green values indicate that the targets were up-regulated under water deficit stress, while red values indicate that the targets were down-regulated under water deficit stress. Bold fold change values indicate negative correlation with Ttu-miR008. FL = Flag leaf libraries; H = Head libraries.
(XLS)

S9 Table Forward primers used in qPCR validation of seven conserved and three novel miRNAs in durum. Each forward primer was designed based on the full sequence of the mature miRNA.
(XLS)

S10 Table Target transcript sequences, primer locations and primer sequences used in qPCR validation of seven selected target genes. (XLS)
