# Supplementary figures and images for: Genome-Wide Identification of MicroRNAs in Leaves and the Developing Head of Four Durum Genotypes during Water Deficit Stress
Source: PLoS One. 2015 Nov 12;10(11):e0142799. doi: 10.1371/journal.pone.0142799 (PMC4643036; doi:10.1371/journal.pone.0142799)

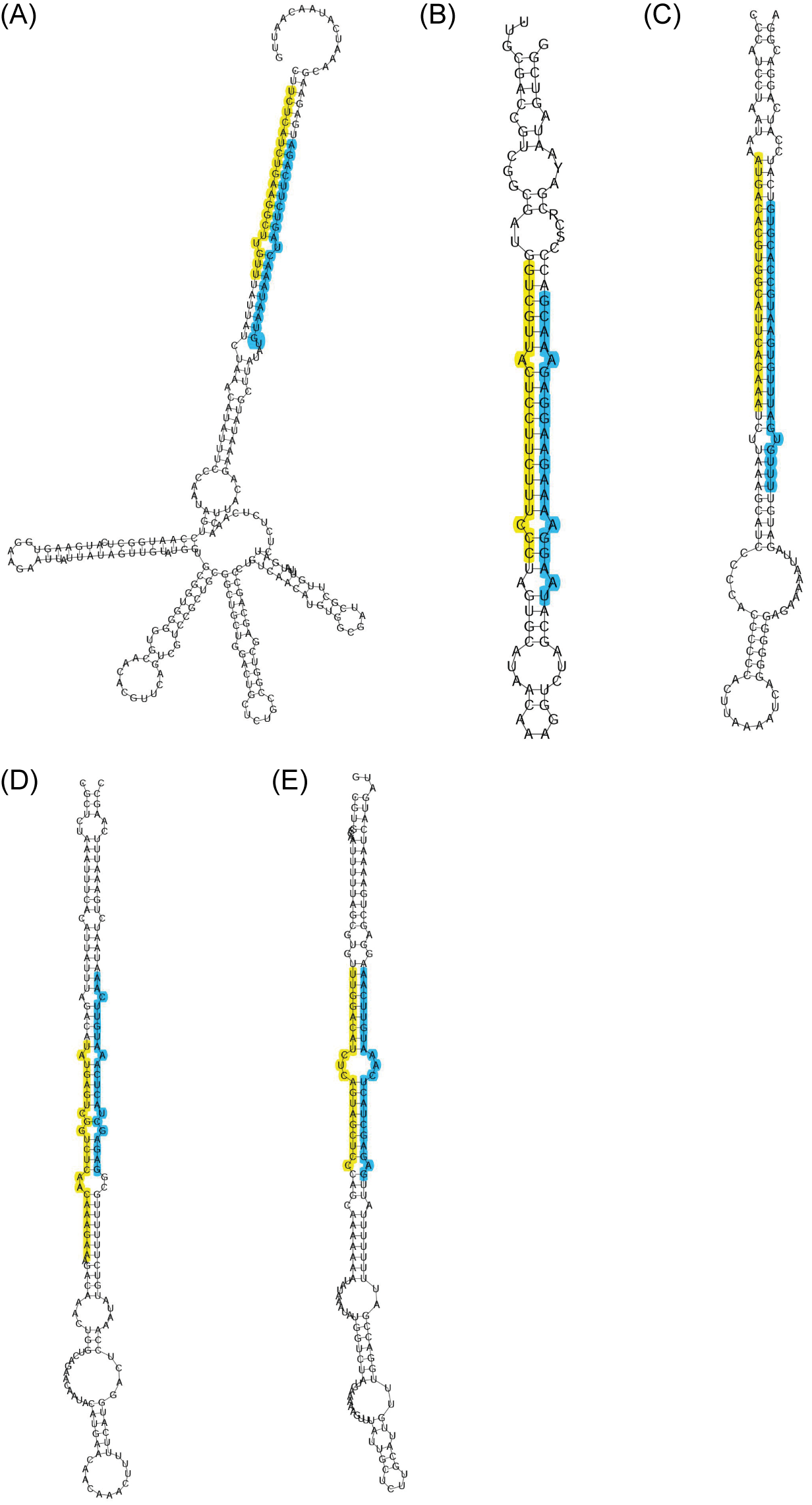

Supplement: S2 Fig — Mature miRNAs are highlighted in blue while miRNA* are highlighted yellow. The secondary structures of the novel durum wheat miRNA hairpins (A) Ttu-pre-miR007, (B) Ttu-pre-miR008, (C) Ttu-pre-miR038, (D) Ttu-pre-miR109, and (E) Ttu-pre-miR119 are shown. (TIF) [file pone.0142799.s002.tif]

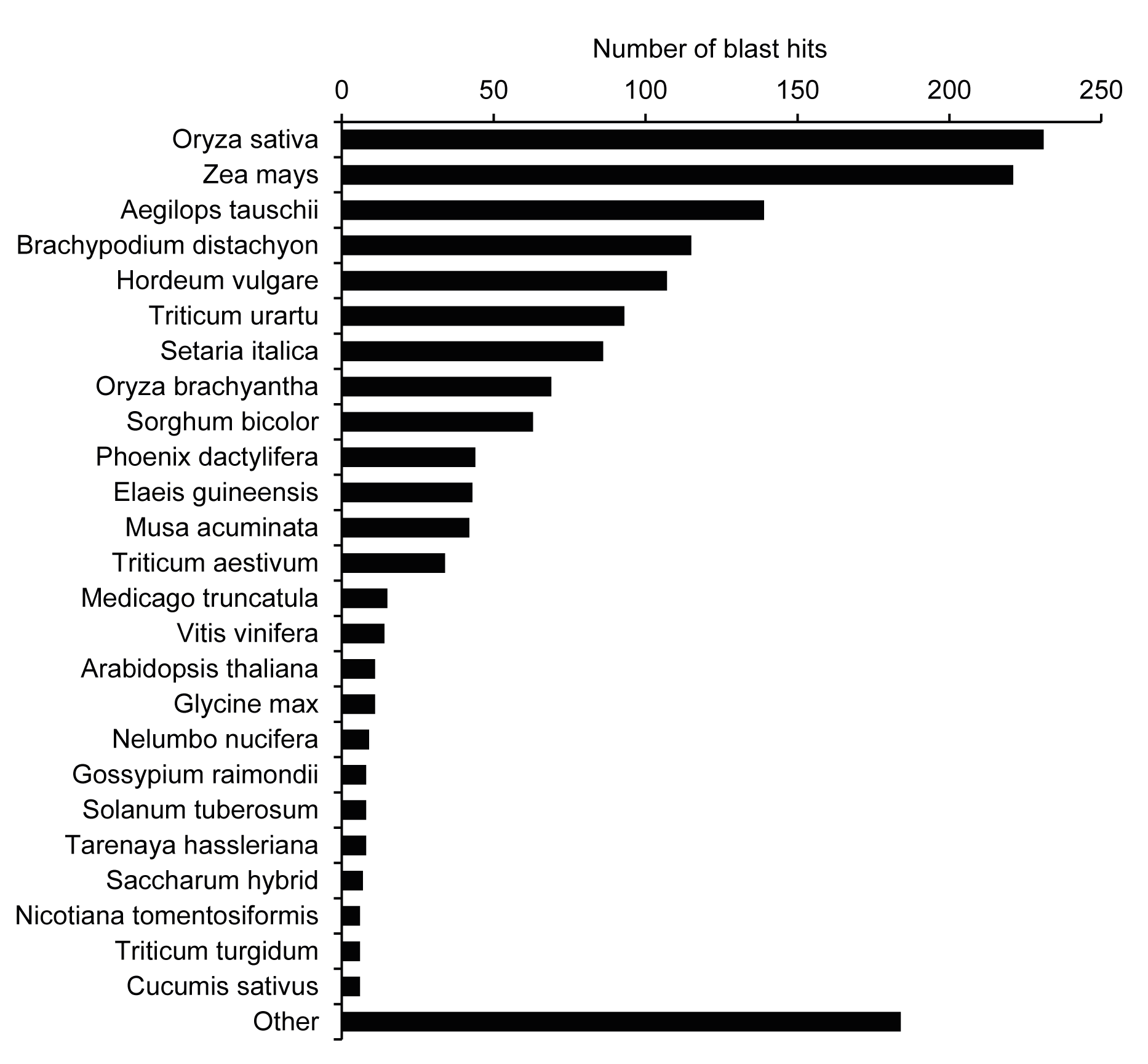

Supplement: S3 Fig — Identified target gene transcripts are searched in the species-specific entries registered in the GO database. Species distribution is based on the number of BLAST hits aligned in each species. (TIF) [file pone.0142799.s003.tif]
